# Nanotechnology-Based Surface Plasmon Resonance Affinity Biosensors for* In Vitro* Diagnostics

**DOI:** 10.1155/2016/2981931

**Published:** 2016-08-10

**Authors:** Riccarda Antiochia, Paolo Bollella, Gabriele Favero, Franco Mazzei

**Affiliations:** Department of Chemistry and Drug Technologies, Sapienza University of Rome, Piazzale Aldo Moro 5, 00185 Rome, Italy

## Abstract

In the last decades,* in vitro* diagnostic devices (IVDDs) became a very important tool in medicine for an early and correct diagnosis, a proper screening of targeted population, and also assessing the efficiency of a specific therapy. In this review, the most recent developments regarding different configurations of surface plasmon resonance affinity biosensors modified by using several nanostructured materials for* in vitro* diagnostics are critically discussed. Both assembly and performances of the IVDDs tested in biological samples are reported and compared.

## 1. Introduction

In the last two decades, the need to obtain early, affordable, and reliable analytical devices for* in vitro* diagnostics has become a key aspect on reaching the quick diagnosis of several pathologies. This is important not only for the safeguard of health and safety of patients but also for reducing the costs of the National Health Systems. An ideal* in vitro* diagnostic device (IVDD) should allow the qualitative and quantitative detection of several pathologies of potential markers in biological fluids and tissues, rather than in a living body [1]. The role played by the IVDDs is (i) to assure a fast screening of populations (action: prevention of diseases); (ii) early and accurate information (action: correct diagnosis); (iii) real-time monitoring (action: evaluation of therapy treatments). For these reasons the European Council fixed strict rules for the characteristics and commercialization of IVDDs (Directive 98/79/EC). In this context, a rigorous classification based on an alphabetical system (Table 1) was made according to the following criteria:

- Manufacturers should specify the use conditions and the risk factor of IVDDs.
- The obtained information should be relevant for a careful diagnosis, taking into account the natural history of diseases.
- The results should affect, positively or negatively, the public/individual health.

The official analytical methods generally employed for biomedical applications are affected by several drawbacks: being time-consuming, costs of analysis, laborious procedures, the need for qualified personnel, and poor availability as point-of-care systems. In the last decades the significant technological progress in several fields such as nanotechnologies, electronics, and biotechnologies as well as the need to have fast, sensitive, and user-friendly devices resulted in the development of several analytical methods. In this field, biosensors can play an important role for some peculiar features. A biosensor is an analytical device where a biotransducer (BT) (e.g., tissues, DNA, antibodies, enzymes, etc.) is coupled to a physicochemical transducer (e.g., electrochemical, optical, piezoelectric, or magnetic). The interaction between the analyte and the biotransducer results in a change of a physical or chemical property detected by the physicochemical transducer and converted into an electrical signal which, opportunely amplified and elaborated, allows obtaining information about the sample under investigation [2].

Among the different types of biosensors, the affinity-based biosensors (ABBs) are considered the most suitable for IVD analysis for some interesting properties: reduced costs of analysis, selectivity, being easy to use, and furthermore the reversible interaction between the biotransducer and the analyte which allows the reuse of the biodevice for multiple analyses. ABBs, characterized by the use of antibodies or aptamers as biotransducers, provide interesting information about the concentration of the analyte and also about the kinetics and thermodynamics of the biotransducer-analyte interaction.

Surface plasmon resonance- (SPR-) based transducers show interesting features compared to other physicochemical transducers, allowing real-time monitoring of biotransducer-analyte interactions, without the need of labeling. The biotransducer immobilized onto the sensor disk surface interacts with the analyte producing a local increase in the refractive index at the metal surface which promotes an SPR signal shift [3, 4].

Besides several advantages of SPR transduction, some drawbacks must be considered such as nonspecific binding phenomena onto the SPR disk which can affect the accuracy of the measurements in biological fluids, the difficulty of immobilization of bioreceptors characterized by steric hindrance, and the inability to detect small molecules characterized by a low molecular weight. The key issue to overcome these drawbacks is a proper sensor chip modification [5].

Recent technological advances in the field of nanotechnology allow the development of new nanostructured materials which have a great impact on the overall performances of biosensors and in particular of SPR-based biosensors. Nanostructured materials can be useful to overcome the problem of a low sensitivity due to small changes in the refractive index at the SPR disk surface arising from analytes with low molecular weight or low concentration values. The nanomodification is important to increase the biotransducer loading thanks to the high surface/volume ratio of these materials as well as amplify the plasmonic signal [6–9]. Graphene and metal nanoparticles (MNPs) show the most suitable characteristics for sensor chip modifications. Graphene coating improves the change of the refractive index at the interface graphene/gold sensor chip, thus modifying the SPR signal associated with the thin metal surface [10]. Metal nanoparticles, in particular, gold and silver nanoparticles (AuNPs or AgNPs), displaying a localized surface plasmon resonance (LSPR), are widely used to enhance the SPR response [11, 12]. In fact, a change in the SPR energy is observed thanks to the coupling of LSP and the SP [13–17]. Some researchers studied the use of AuNPs as amplifier labels for biorecognition events [18, 19], biocatalytic transformations [20, 21], or sensitive detection of low molecular weight compounds [22, 23].

The enhancement of the overall performances achieved with the nanomodification of the SPR-based biosensors results is of extreme importance for their application as IVDDs [24, 25].

In this review, we describe and discuss the potential use of nanomodified SPR-based biosensors for IVD applications. In particular, three different SPR configurations have been taken into account: (a) bulk SPR; (b) localized SPR; (c) SPR imaging.

**Figure 1:**
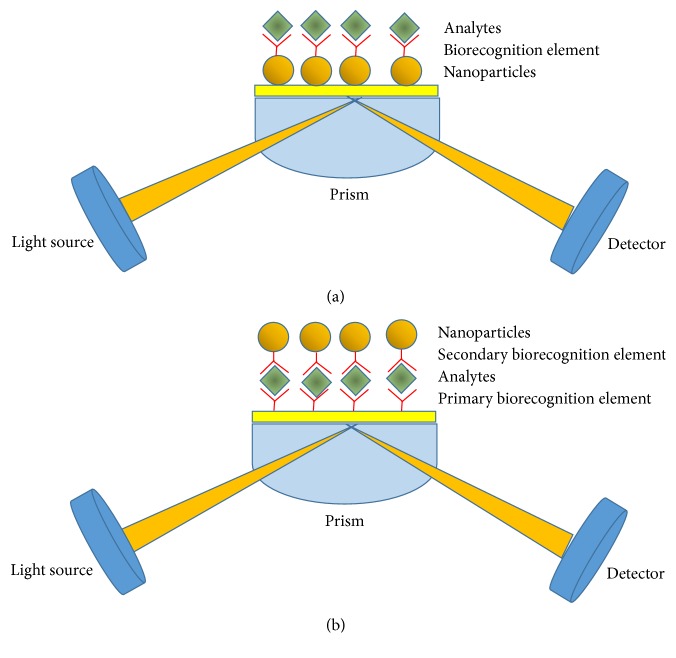
Scheme of bulk SPR with (a) nanoparticles incorporated onto the surface and (b) sandwich “assay” with nanoparticles amplification.

**Figure 2:**
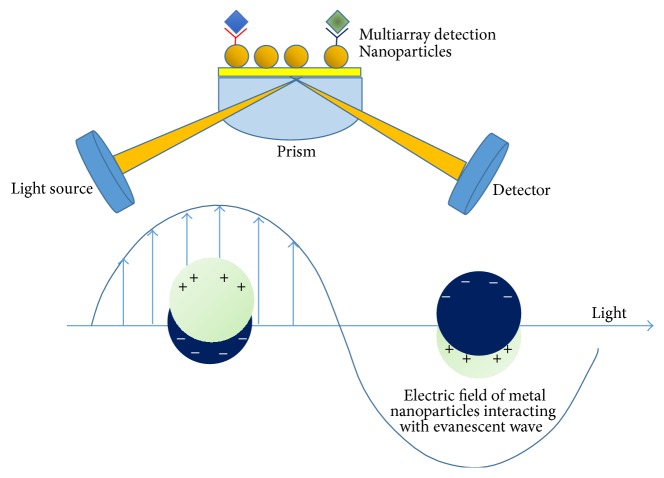
Scheme of localized SPR by using metal nanoparticles as amplification system to improve the IVD devices thanks to the local electric field/surface plasmon interaction.

**Figure 3:**
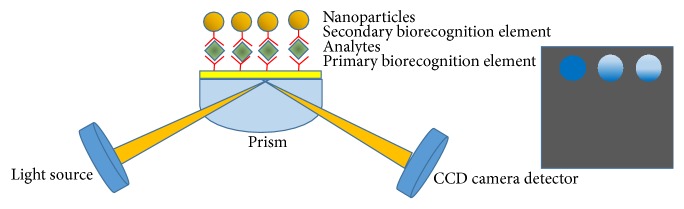
Scheme of SPR imaging.

- *Bulk SPR*. This technique is based on the detection of small changes in the refractive index on thin metal films arising from the binding of analytes with a specific biotransducer through the change in the resonance angle. The use of nanoparticles increases the bulk SPR sensitivity with a consequent reduction of detection limit through two different mechanisms: (i) the particular interaction between the electrical field developed from metal nanoparticles and the evanescent wave due to the plasmon surface excited from light source [26]; (ii) the bioconjugation with a biological recognition element, antibody or aptamer, which enhances the molecular weight, as the SPR signal is directly proportional to the mass immobilized on the surface. In the first case the metal nanoparticles are incorporated onto or into the metallic surface (Figure 1(a)), while in the second case they are utilized in the so-called “sandwich assay” format (Figure 1(b)) where the MNPs, conjugated with secondary antibodies or aptamers, are captured by the sensor surface subsequent to the binding of the target analyte to the primary biorecognition elements.
- *Localized SPR*. When the charge density oscillation is confined to metallic nanoparticles or nanostructures the localized surface plasmon resonance (LSPR) phenomenon is generated. When this occurs, there is an incident light source that induces both light scattering and a strong absorbance, with a consequent enhancement of the local electromagnetic field [27] (Figure 2). The frequency and the absorption bands depend on the type of material (e.g., gold, silver, and platinum) but also on the shape and size distribution that realize particular conditions onto the surface to enhance the SPR signal. Therefore the sensitivity and selectivity can be modified by changing the shape, size, and material composition of MNPs to achieve ultrasensitivity and ultraselectivity thanks to the novel nanoconfigurations [28]. Moreover, LSPR allows a simultaneous detection of different biomarkers with different concentrations, thus enabling high-throughput screening in the array format [29].
- *SPR Imaging*. One of the last SPR approaches is the surface plasmon resonance imaging (SPRi), a combination of SPR and a spatially resolved measurement (Figure 3). This kind of resolution, by using a two-dimensional charge-coupled device (CCD) detector array, allows monitoring the biomolecular interactions to determine kinetic and analytical parameters. The output of this instrument is an image with different spots where the light intensity is proportional to the different analyte concentration that interacts with the biorecognition element. Thanks to this 2D-detector it is possible to detect small SPR signal variation [30].

By comparing SPRi with a conventional bulk SPR, the advantages of SPRi are the possibility of detecting more biomarkers at the same time, with an enhanced sensitivity and selectivity. Colloidal nanoparticles (NPs) improve the sensitivity of SPRi to ensure rapid, proper, and sensitive analysis. The focus point of SPRi is the optical part of the instrument where the light is addressed to different spots, properly modified, to produce an SPR image thanks to the reflected light [31].

Finally, in Table 2 the main advantages and drawbacks of bulk, localized, and imaging SPR analytical techniques are reported.

## 2. Nanomodified SPR-ABBs Applications for* In Vitro* Diagnostics

A detailed description of recent applications of nanomodified SPR biosensors for IVD applications is reported.

### 2.1. Bulk SPR

To enhance the SPR sensitivity metal nanoparticles are often used; this is accomplished throughout different approaches: (i) nanoparticles directly incorporated into the surface, (ii) entrapment of nanoparticles in structures for spacing control, (iii) sandwich assay format by functionalization of nanoparticles, (iv) employment of magnetic nanoparticles, and (v) enzyme-conjugated nanoparticles.

In the first papers, MNPs were directly incorporated into the electrode surface, as reported in the following works where biosensors are described for several biomarkers. Several examples deal with different arrangements employing AuNPs to enhance sensitivity towards low molecular weight molecules: Jung and coworkers developed an SPR biosensor for the detection of prostate-specific antigen (PSA), a cancer biomarker, by immobilizing AuNPs onto a SiO_2_ layer on a gold electrode and the results were compared with those obtained with both an unmodified gold surface and a SiO_2_ layer on a gold surface. The detection limit of the biosensor was 0.1 ng/mL for PSA, which is comparable to the values obtained with standard ELISA tests with sensitivities in the range 0.1–10 ng/mL [32].

Li et al. realized an SPR biosensor for the determination of ischemia modified albumin (IMA), a biomarker capable of reflecting myocardial ischemia condition, by assembling anti-IMA onto an AuNPs modified gold chip. Here IMA was detected at 10 ng/mL and no interferences were reported. The modified biosensor showed also high sensitivity thus providing an effective new approach for a direct assay of IMA [33].

Progesterone, an important reproductive hormone, was detected by Yuan et al. by an ultrasensitive SPR inhibition immunoassay using a mixed self-assembled monolayer (mSAM) surface to keep the AuNPs close to the sensing surface. Progesterone was conjugated to ovalbumin with an oligoethylene glycol linker to form a protein conjugate, immobilized onto the mSAM surface, and then detected with a low detection limit of 4.9 ng/mL. Furthermore, the technique uses inexpensive reagents, and the stable mSAM surface provides multiple reuses [34].

An innovative approach for the employment of AuNPs is the possibility of AuNPs entrapment in structures for spacing control. In fact, the spacing between particles and surface was found to have a significant influence on the enhancement of the SPR signal [35]. One possibility for achieving this enhancement is the encapsulation of the particles into dendrimers. An SPR biosensor for detection of insulin in human serum using a bifunctional hydroxyl/thiol-functionalized fourth-generation polyamidoamine dendrimer to encapsulate AuNPs has been reported by Frasconi et al. The resulting AuNPs dendrimer-modified surface is a good support for insulin immobilization. The immunosensor based on an indirect competitive immunoassay principle showed high sensitivity, specificity, and stability for detection of insulin in human serum with a detection limit of 0.5 pM [36].

Another option is the entrapment of AuNPs into molecularly imprinted polymers (MIPs). An SPR sensor chip for the detection of dopamine using a molecularly imprinted polymer with embedded AuNPs, immobilized on a decanethiol-modified Au chip, was described by Matsui et al. The sensing principle is based on swelling of the imprinted polymer gel triggered by the analyte binding within the polymer gel, which increases the distance between AuNPs embedded in the film resulting in a change of the SPR signal. Both the analyte binding process and the consequent swelling are reversible processes thus allowing the repeated use of the presented sensor chip [37].

Better results were obtained when MNPs are conjugated with antibodies or aptamers in the “sandwich assay” format, as reported in the following papers.

Choi et al. realized an ultrasensitive SPR affinity device based on the conjugation of AuNPs and the polyclonal antibody for PSA detection. The monoclonal antibody is highly oriented onto the surface thanks to the first layer formed by rProtein G deposited on the surface by self-assembled monolayer. The highly oriented antibody immobilized on the surface and the conjugation with AuNPs-antibody allowed the detection of PSA in the femtomolar range (detection limit 300 fM), with a good linearity range, thus increasing 1000-fold the sensitivity of PSA detection compared to previous works [39].

Another example of a sandwich configuration is the AuNPs modified SPR biosensor for the detection of human cardiac myoglobin (cMb) in serum based on a reverse sandwich assay, described by Gnedenko and coworkers. A first monoclonal anti-cMB antibody was covalently immobilized on the sensor surface and then AuNPs were covalently immobilized to the second monoclonal anti-cMb antibody. The limit of detection of cMb in a human blood serum sample was found to be as low as 10 pM with an intra-assay coefficient variation of less than 3% [40].

Špringer and Homola developed an SPR affinity biosensor to detect low levels of carcinoembryonic antigen (CEA) in human blood plasma based on a new biofunctionalization of AuNPs. The AuNPs were functionalized either with streptavidin, to provide high affinity for the biotinylated secondary antibody, or with bovine serum albumin, to minimize the nonspecific adsorption of proteins during the analysis of complex samples like blood plasma. The basic concept of this approach is the sandwich assay format using a primary antibody immobilized on the surface, the antigen CEA with different concentrations, and the biotinylated secondary antibody bound with the bio-AuNPs. This approach makes the detection of CEA at concentrations of 0.1 ng mL^−1^ possible, much lower than normal physiological levels [41].

In a successive work, Špringer and coworkers also studied the effects of AuNPs on the response of an SPR sensor for the detection of a carcinoembriogenic antigen (CEA). AuNPs were functionalized with neutravidin (N-AuNPs) in a sandwich assay format in order to enhance the sensor response to a biotinylated secondary antibody against CEA. The authors demonstrated that the sensor response enhancement was determined by two factors, both depending on the size of AuNPs: sensor sensitivity to N-AuNPs surface density and the ability of N-AuNPs to bind functionalized sensor surface [35].

In other cases, the “sandwich assay format” can be realized by functionalizing the MNPs with aptamers, artificial single-stranded DNA, or RNA sequences with extremely high affinity and specificity for specific targets. Compared to traditional antibodies, aptamers have unique properties such as low immunogenicity, ease of chemical modification, and high structural stability and flexibility. Therefore MNPs-based SPR aptasensors combined with sandwich assays represent an alternative strategy to detect proteins with enhanced sensitivity and selectivity.

A sandwich format with aptamer/thrombin/aptamer-AuNPs system on an SPR sensor for subnanomolar detection of thrombin was described by Bai et al. The sensor mechanism is based on the simultaneous binding of thrombin to different aptamers at two exosites. One thiol-modified thrombin aptamer was immobilized on AuNPs via Au-S bonding. The other biotinylated thrombin aptamer was grafted onto streptavidin pretreated SPR gold film through biotin-streptavidin recognition. Thrombin is able to create a double aptamer sandwich structure on the SPR gold surface which allows a large enhancement of the SPR signal. Thrombin was detected in the range 0.1–75 nM with a detection limit of 0.1 nM, 5-fold lower than that obtained in a direct detection format without AuNPs [43].

Jang and coworkers developed a novel approach for the ultrasensitive detection of the brain natriuretic peptide (BNP), a short peptide recognized as a cardiac biomarker, based on a “sandwich assay” DNA aptamer-biomarker-antibody. This method was realized by chemical binding of DNA aptamer on the surface by self-assembling, followed by the affinity recognition with the biomarker, BNP, and the antibody. The antibody was labeled with gold nanocubes (AuNCs) in order to enhance the SPR signal by increasing the mass immobilized onto the surface. The combination of AuNCs labeling and the particular sandwich assay format allows reaching detection limits in the attomolar range, avoiding the nonspecific adsorption of proteins [44].

Also gold nanorods can be used in the “sandwich assay” format to detect concentrations in the femtomolar range. Law et al. proposed a gold sensor chip modified with 3-mercaptopropionic acid (MPA) self-assembled monolayer that allows the immobilization of monoclonal antibody for the detection of tumor necrosis factor alpha (TNF-*α*) antigen. The immunoreaction between the antigen, TNF-*α*, and the secondary antibody bioconjugated with gold nanorods is an ultrasensitive method to increase the sensitivity. The detection limit of TNF-*α* antigen was 0.5 ng mL^−1^, much lower than the physiological value of this antigen [42]. Other strategies to reach detection limits in the subattomolar range involved the use of dual nanoparticles. Baek et al. used two different AuNPs shapes, nanorods (NRs) and quasi-spherical nanoparticles (qsNPs) to realize an SPR affinity biosensor for thrombin (Thr) detection. The modification was carried out with a covalent binding of antithrombin using a mixed self-assembled monolayer. After the affinity recognition of the antigen, the interaction with a DNA pair modified with a polyadenine tail took place. The following step was the matching with the complementary polythymine aptamer (pT) previously modified with qsNPs, followed by a dual interaction with another polyadenine aptamer, modified with NRs for a double SPR signal enhancement. The sensor chip allowed detecting thrombin concentrations as low as 0.1 aM, with a 10-fold sensitivity enhancement compared with a simple NPs modification [45].

Rolling circle amplification (RCA) is an advanced molecular amplification technique, recently used to amplify the SPR signal. He et al. detected human thrombin using SPR and quartz crystal microbalance (QCM) sensing platforms in combination with aptamer-based rolling circle amplification and bio-bar-coded AuNPs enhancement. Thrombin was detected in the range 1 aM–0.1 pM with an excellent detection limit of 0.78 aM. In the absence of amplification the detection limit for thrombin was about 0.1 nM, thus showing 4 orders of magnitude improvement in the presence of RCA [46].

Also magnetic nanoparticles (MNPs) can be used as labels. They have been receiving increasing attention because of their high refractive index and high molecular weight which allow them to be successfully used as amplifying agents in SPR biosensing design. Wang et al. demonstrated that the addition of antithrombin aptamer-Fe_3_O_4_ MNPs conjugates greatly enhances the sensitivity of an SPR sensor for the detection of thrombin. Thrombin was captured by immobilizing antithrombin aptamer on SPR gold film with a very low detection limit of 0.017 nM, thus confirming that MNPs are a powerful sandwich element and an excellent amplification reagent for SPR-based sandwich assays. The limit of detection obtained was lower than that obtained for the sandwich assay with AuNPS, but higher than with the use of rolling circle amplification [47].

Liang et al. used the core-shell Fe_3_O_4_-Au magnetic nanoparticles (MNPs) as amplification method for the detection of human *α*-fetoprotein (*α*-FET). The gold sensor chip was modified with a film formed by 3-mercapto-1-propanesulfonate/chitosan-ferrocene/AuNPs, followed by the covalent binding of primary antibody and bovine serum albumin (BSA) to avoid the nonspecific adsorption of proteins. Successively, there was the affinity recognition with the target and secondary antibody bioconjugated with the core-shell Fe_3_O_4_-AuNPs. The calibration curve of *α*-fetoprotein was obtained in the range 1–200 ng mL^−1^, with a LOD of 0.65 ng mL^−1^ [48].

Recently, superparamagnetic nanobeads were used for SPR signal amplification. Soelberg and his coworkers developed a method for rapid purification, concentration, and detection of Staphylococcal Enterotoxin B (SEB) using antibody-coated superparamagnetic nanobeads. The sensitivity was 10-fold enhanced with a detection limit of 100 pg mL^−1^. The “sandwich assay” approach was provided with the monoclonal antibody immobilized onto the surface and the secondary antibody bioconjugated through the biotin-streptavidin interaction between the antibody and the magnetic beads [49].

Another approach for SPR signal amplification concerns enzymatic precipitation, as described by Cao and Sim: the authors described a method based on the synthesis of an AuNPs-enzyme conjugate for the amplification of the SPR-based detection of anti-glutamic acid decarboxylase antibody (anti-GAD), an autoantibody present in the serum of insulin-dependent diabetes mellitus patients. The AuNPs were covalently conjugated with horseradish peroxidase (HRP) and anti-IgG antibody to form an enzyme-immunogold complex. The combination of AuNPs and enzyme precipitation is the strategy used for signal enhancement. Anti-GAD Ab was detected at a concentration as low as 0.03 ng mL^−1^ [69].

The main characteristics of the biosensors reviewed in this section are summarized in Table 3.

### 2.2. Localized SPR

One of the main important challenges in the LSPR performances enhancement is the improvement of sensitivity and selectivity in complex media analysis. As for bulk SPR, the LSPR-based ABBs sensitivity is improved by using the sandwich assay configuration, through the bioconjugation with antibodies or aptamers as biotransducer.

Mayer and coworkers reported the development of gold nanorods based LSPR biosensor. The nanorod surface was functionalized with a self-assembled monolayer and bound to the antibody using carbodiimide cross-linking. The interactions with a secondary antibody were studied by monitoring the shift in the LSPR signal resulting in a little binding of the nonspecific secondary antibody. These findings demonstrated that the nanorod-based LSPR sensor could be able to perform the real-time monitoring of dynamic interactions in the same way of bulk SPR, but with much simpler instrumentation required [28].

Haes et al. realized an LSPR biosensor for the determination of biomarker of Alzheimer's disease by measuring the interactions between amyloid-derived diffusible ligands (ADDL) and the anti-ADDL antibody, involved in Alzheimer's disease. Alzheimer's disease (AD) is a progressive mental disorder disease widely diffused in the world; since a specific cure is unavailable at present, an early diagnosis of AD is a crucial point for the existing drug treatments. Based on a sandwich assay format the proposed biosensor showed quantitative binding information for both antigen and secondary antibody detection allowing the determination of ADDL concentration which is higher in disease patients in comparison to the control patients [50].

Park et al. realized an LSPR biosensor using multispot gold-capped silica nanoparticle array chip for detection of avian influenza (A) virus, an infectious viral disease. A metal binding polypeptide (GBP)-fusion protein was bound onto the gold substrates by means of specific interactions allowing immobilization of proteins in bioactive forms onto the gold surface suitable for immunosensors applications. Following this procedure, an ultrasensitive detection of avian influenza virus was successfully achieved up to 1 pg mL^−1^ [51].

A highly sensitive LSPR immunosensor was proposed for the detection of HIV-1 virus based on the realization of a nanopattern of circular gold nanodots which was electrodeposited onto an indium tin oxide coated glass substrate [52]. The resulting modified surface was then functionalized with the HIV-1 antibody fragments allowing the measurements of various concentrations of HIV-1 particles with a detection limit of 200 fg mL^−1^, 10-fold higher than that of previously reported virus detection method based on LSPR. This approach could be useful for the development of biodevices for the analysis of several viruses' detection.

Chen et al. realized a simple and affordable LSPR-based device for the highly sensitive detection of both extracellular adherence protein (EAP), found on the outer surface of the bacterium* Staphylococcus aureus* (*S. aureus*), and prostate-specific antigen (PSA). The EAP, a protein of 60 kDa excreted by* S. aureus*, has affinity for both eukaryotic cells and the surface of* S. aureus* itself. The sensor chip surface was modified by self-assembling to immobilize the specific antibodies for EAP and PSA detection, which are anti-EAP and the monoclonal anti-Kallikrein 3 (anti-Klk3) for anti-PSA. This device showed a high sensitivity, with a LOD of 8 nM for EAP and 1 pM for PSA. These experiments can be considered as a proof of concept for the development of high-performance biosensing platform for point-of-care diagnostics applications [53].

Zhao and coworkers developed an LSPR biosensor for the detection of serum squamous cell carcinoma antigen (SCCa), a well-known tumor biomarker important for the early diagnosis of cervical cancer diseases, which is one of the most widespread cancers in women. The LSPR biodevice is based on the use of a triangle-shaped silver nanoparticle array obtained by nanosphere lithography method, with monoclonal anti-SCCa antibodies immobilized onto the resulting surface. Several concentrations of SCCa were measured both in buffer and in human serum with a linear detection range of 0.1–1.000 pM with a LOD of 0.125 pM. This device could be useful as a potential alternative to the traditional technique commonly used in the diagnosis of cervical cancer diseases [54].

Aćimović et al. developed an application of a parallel LSPR biodevice with enhanced analytical performances (high-throughput, fast, and real-time monitoring) for the analysis of two well-known cancer markers: human alpha-feto-protein (AFP) and PSA. The system device enables the simultaneous, real-time analysis of 32 sensing spots distributed across eight independent microfluidic channels. Each spot is obtained by the deposition of gold nanorods onto a glass substrate in line. The isolation among each array is achieved by a polydimethoxysilane (PDMS) microfluidic interface controlled by micromechanical valves. The detection of the cancer markers can be performed in a dual configuration: (a) direct format; (b) sandwich format by using a secondary antibody. This system allowed the detection of the two cancer markers down to concentrations of 500 pg/mL in a real serum matrix [55].

Yuan et al. carried out a study to develop a novel LSPR biosensor for detecting human epididymis secretory protein 4 (HE4) biomarker in blood samples for the early diagnosis of ovarian cancer. On the surface of silver nanoparticles, obtained by nanosphere lithography method, was immobilized the anti-HE4 antibody by using an amine coupling method. The performances of the device allow a fast, accurate, and reproducible analysis with a linear range ranging from 10 pM to 10,000 pM and a LOD of 4 pM [56].

Lai et al. realized an almost similar approach based on the same Ag nanoparticles modification of the sensor surface for the detection of microalbuminuria in urine samples. Screening microalbuminuria could identify the patients that are at high risk for cardiovascular events with pregnancy-induced hypertension and diabetes mellitus. An amine coupling procedure was employed to immobilize the anti-human albumin antibody on the sensor surface. The biosensor displayed short analysis time (about 40 minutes), a detection limit of 1 ng/mL, and a wide dynamic range of 1 ng/mL to 1 *μ*g/mL [57].

Duan and coworkers developed a nanobiosensor based on LSPR for detecting p53 mutation. p53 is a key tumor suppressor that plays crucial roles in the induction of cell cycle arrest and apoptosis as a consequence of DNA damage. p53 mutations, especially those occurring in L2 and L3 zinc binding domains, are strongly associated with radiotherapy and chemotherapy-resistance. The detection of p53 mutation is of extreme importance to speed up the diagnosis of tumor patients. The DNA probe was designed to recognize the target sequence and immobilized on the chip surface by a covalent coupling method using amine-group ligands. Synthetic oligonucleotides or PCA products were amplified from genomic DNA taken from blood samples and hybridized with the immobilized probe. The detection limit of the sensor was 10 nM with a dynamic range from 10 nM up to 10 *μ*M. The sensor was able to discriminate differences in measuring signals between wild-type and mismatched p53 DNA, demonstrated to be able to discriminate effectively against single base mutations. The results obtained with this biosensor suggested that it can represent an attractive alternative for clinical detection of genetic mutations [58].

Gu et al. proposed an automatic, robust, and high-throughput method to study the resonance scattering light of single plasmonic nanoparticles in the dark-field image for intracellular analysis, thus demonstrating that this approach allows obtaining detailed and reliable information for monitoring the distribution of NADH in cancer cells and estimating the efficiency of cancer drugs [59].

The main characteristics of the biosensors reviewed in this section are summarized in Table 4.

### 2.3. SPR Imaging

An important strategy to improve the reverse chemical genetics was the small-molecule microarray technology development to observe every possible small-molecule-protein interaction. In the last years several procedures were developed to avoid the nonspecific adsorption of proteins and the structural adsorption of small molecule not due to chemical binding [70–73]. To observe interactions between small molecules and proteins, these latter were labeled with a fluorescent molecule, but sometimes there are incompatibilities reducing the protein functionalities [74]. An alternative procedure to detect the interaction and to determine kinetic, thermodynamic, and affinity parameters without labeling is the surface plasmon resonance imaging (SPRi) [75–77].

SPRi has been used to characterize thin organic and biopolymer films at metal interfaces in a spatially resolved mode. This approach enables the measurement of interactions between unlabeled biological molecules and surface-bound species in an array format. Hence, SPR imaging is thought to be well-suited not only for DNA and protein microarrays but also for small-molecule microarrays [78–81]. Indeed, a variety of small molecules have been introduced on SPR biosensors for analyzing interactions between these molecules and proteins; however, the immobilization protocol had to be optimized for the respective small molecules [82–84].

In this section, many approaches based on the nanoparticles were presented with particular attention to the surface modification and the nanoparticle functionality. In fact, they work not only as a labeler but also as a simple amplifier because of the interaction with evanescent wave onto the surface [85–87]. We make a comparison between the following examples focusing on the sensitivity, the modification, and the class of biomarker. Mainly, the biosensors based on SPR imaging can be divided into two groups even if the major part is formed by DNA/RNA aptamer affinity platform, as reported in Table 5.

Sendroiu et al. developed a DNA multidetection array based on the SPR imaging for the detection and identification of nucleic acids. The gold surface was modified by using amine-modified ssDNA elements in order to create four generator elements, where the ssDNA have a complementary sequence for the transcription of RNA with a partially complementary sequence; around them there are eight detector spots, where the ssDNA, modified with 13 nm gold nanoparticles in order to enhance the SPR response, were partially complementary with the ssRNA transcripts. On the surface there are two positive and two negative controls to verify the possible nonspecific adsorption of proteins. In summary, this modification allows detecting the RNA transcripts in the concentration range from 1 fM to 10 pM [60].

A new multidetection method to detect short RNA by using enzymatic and modified silica nanoparticles (SiNPs) amplification to enhance SPR imaging signal was realized from Zhou and his coworkers. SiNPs were functionalized with 5′-phosphorylated single-stranded DNA (ssDNA) coupled with T4 RNA ligase to capture different single-stranded RNA (ssRNA) from a target solution. This procedure by using modified SiNPs presents several advantages: (i) larger surface area exposition to target solution containing ssRNA; (ii) increase of ssRNA diffusion rate thanks to modified SiNPs; (iii) SPR imaging signal enhancement by using SiNPs. The preliminary measurements demonstrate that this detection method can be used to investigate multiple ssRNA sequences at concentrations as low as 100 fM in 500 *μ*L [61].

Fang et al. developed a new approach for the miRNAs detection by using a locked nucleic acids (LNA) modified surface to monitor concentration down to 10 pM with the SPR imaging [62]. In the first step, there is the complementary interaction between the LNA and the miRNA with 3′-OH end group disposable to bind a polyadenine tail by pA-polymerase. Successively, the recognition interaction with polythymine tails modified with gold nanoparticles in order to amplify the SPR imaging signal to allow the detection of low miRNA concentrations was carried out. The multiplexed detection of miRNAs with DNA microarrays is a particularly appealing method for miRNA profiling in biological samples.

Using this technique, Corn's research group has been able to detect simultaneously three different miRNAs present in total RNA sample extracted from mouse liver. A four-component microarray was constructed containing three LNA probes designed to bind to miR-16, miR-122b, and miR-23b miRNA sequences, with a DNA probe used as a negative control. The concentration of the different miRNA species in the solution has been calculated from calibration curves of the SPRi response signal obtained using synthetic analogs of the target sequences, and the amount of miRNAs in the total RNA sample was estimated to be 20 fM, 50 fM, and 2 pM for miR-16, miR-23b, and miR-122b, respectively. The detection limit of this amplified SPRi methodology was found to be 5 attomoles, which is at least 50 times more sensitive than the fluorescent-based microarray detection methods.

The variety of SPR imaging biosensing applications can be expanded by enzymatic manipulation of DNA microarray elements, and the sensitivity of detection can be enhanced by the use of oligonucleotide immobilized onto a gold nanoparticle surface. Gifford et al. developed a new method where a template-directed polymerase extension of a surface array element and nanoparticle-enhanced detection of the reaction product were coupled. This technique allows detecting concentrations of the polymerase in the linear concentration range of 10–100 attomoles through the DNA target produced. This sensitivity would allow for the detection of a specific DNA target that is present in low amounts in a sample and with partially unknown sequence. One application of this method would be to identify the presence of the aberrantly recombined DNA sequences, such as those found in the fragile sites of chromosomes [63].

A sensitive method based on nanoparticle-enhanced surface plasmon resonance imaging to detect single nucleotide polymorphisms (SNPs) in genomic DNA was realized from Li and his coworkers. Sequence-specific surface reactions of the enzyme* Taq* DNA ligase were used to investigate SNPs, and the presence of ligation products on the DNA microarray elements was detected using SPRi through the hybridization adsorption of complementary oligonucleotides labeled to gold nanoparticles. The concentration of 1 pM was successfully identified thanks to the gold nanoparticles amplification that allowed improving the SPRi sensitivity [14, 15].

This sensitivity is sufficient enough for performing multiplexed SNP genotyping by using multiple PCR amplicons and should also allow for the direct detection and identification of SNP sequences from 1 pM unamplified genomic DNA samples with this array-based and label-free SPRi methodology. As a first example of SNP genotyping, three different human genomic DNA samples were screened for a possible point mutation in the* BRCA*1 gene that is associated with breast cancer.

Recently Sato et al. reported that gold nanoparticles (GNPs) with fully matched duplexes on their surfaces are selectively deposited onto walls of poly(dimethylsiloxane) (PDMS) microchannels at high salt concentrations. In this study, the surface plasmon resonance (SPR) imaging technique was used to investigate this phenomenon for improvement of detection sensitivity and elucidation of the phenomenon. The microchip was realized by bonding a surface-patterned PDMS plate and a gold thin film-deposited glass substrate. Probe oligonucleotide-modified GNPs were hybridized with target oligonucleotides to make fully matched or single-base-mismatched duplexes. The hybridized GNP solution was mixed with a NaCl solution in a Y-shaped microchannel [64].

SPR imaging allowed controlling the GNP deposition but also discriminating the targets with limit of detection of 32 nM (19 fmol) without temperature control in 5 min. Therefore, in combination with a portable SPR device, the proposed method is promising for point-of-care testing of single nucleotide polymorphisms [88, 89].

For a proper DNA detection the technologies available today are based on the combination of labeled probes and target sequences amplified by polymerase chain reaction PCR. The elimination of PCR and labeling is the focus point in order to develop a new biosensor for early, proper, and low-cost analysis [65]. In this work D'Agata et al. describe the results obtained in the ultrasensitive detection of nonamplified genomic DNA. Certified reference materials containing different amounts of genetically modified DNA by using a detection method which combines the nanoparticle-enhanced surface plasmon resonance imaging (SPRi) biosensing to the peptide nucleic acids (PNAs) improving selectivity and sensitivity in targeting complementary DNA sequences were realized. The method allowed obtaining a 41 zM sensitivity in targeting genomic DNA even in the presence of a large excess of noncomplementary DNA.

Yao et al. reported the development of oligonucleotide (ODN) coating gold nanoparticles (Au–NPs) amplification in a sandwich assay of ODN or polynucleotide by a flow injection surface plasmon resonance (SPR) imaging. The disk surface was modified by spreading a carboxylated dextran film to minimize the aspecific adsorption of ODN coating Au–NPs. The synergic action of oligonucleotide enhanced loading and AuNPs plasmonic effect results in a signal amplification measured by the bicell detector on the SPR with a notable DNA LOD of 2.1 × 10^−20^ mol. This biosensor showed high reproducibility and specificity and could be employed also for the p53 cDNA (a cellular tumor antigen) analysis [66].

Wood and her coworkers [67] reported a single step polymerization of polydopamine (PDA) films onto a thin film gold substrate and then employed it in the development of DNA microarrays for the sensitive detection of biomolecules with nanoenhanced surface plasmon resonance (SPR) imaging. The PDA multilayer thicknesses, ranging from 1 to 5 nm, were characterized by means of scanning angle SPR and AFM techniques, and a 1.3 ± 0.2 nm PDA multilayer thickness was chosen as optimal for the SPR imaging measurements. The DNA microarrays were obtained by coupling the amine-functionalized single-stranded DNA (ssDNA) oligonucleotides with PDA-modified gold thin film microarray element. Then they were employed in the measurements of DNA hybridization adsorption and protein-DNA binding [90–92]. The PDA multilayer showed good resistance to the nonspecific binding of biomolecules. The results obtained suggest the possible use of these ssDNA microarrays for the detection of miRNA.

An affinity immunoreaction sandwich by using CCD detector to capture the SPR image at visible wavelength was realized from Paul and his coworkers. The surface modification started with a self-assembling of 11-mercaptoundecanoic acid in order to immobilize the primary anti-immunoglobulin G. Successively, BSA injection was performed to avoid the nonspecific adsorption of proteins onto the surface. The next step was the immunoreaction with the antigen, immunoglobulin G (IgG), and the secondary antibody labeled with silver nanoparticles to increase the SPR signal.

The device allows investigating the IgG concentration in the linear range of 6.66–660 nM, ensuring a lower detection limit of 2 nM with the electroaddressing of the secondary antibody, and a lower detection limit of 1 nM in the presence of other low molecular weight proteins [68].

The main characteristics of the biosensors reviewed in this section are summarized in Table 5.

## 3. Conclusions

The most recent advances about the employment of nanostructures in SPR allowed greatly enhancing the analytical performances of* in vitro *diagnostics. In particular, bulk SPR devices took great advantages from the use of these materials getting to lower the limit of detection to fractions of ng/mL and sometimes to concentrations levels lower than 0.5 pM. This was made possible thanks to different approaches such as the encapsulation of the particles in dendrimers and the development of sandwich assay formats that in some cases enable detecting concentration as low as in the femtomolar range. On the other hand, the detection limits in subattomolar range can be reached by different strategies based on the use of dual nanoparticles.

The localized SPR-based methods can be used to monitor real-time dynamic interactions in a similar manner to bulk SPR, but with much simpler instrumentation; simple and cost effective LSPR-based devices proved to be useful for detection of extracellular proteins at attomolar levels.

Lastly, the SPR imaging-based methods have been shown to represent an alternative procedure to analyze interactions of either DNA, protein microarrays, or small-molecule microarrays and to determine kinetic, thermodynamic, and affinity parameters thereof without labeling. Amplified SPRi methodologies accomplished detection limits as low as attomolar levels, which is at least 50 times more sensitive than the fluorescent-based microarray detection methods.

The outlook of SPR techniques employed as IVDDs will go in two directions: (i) miniaturization in order to obtain portable and reliable devices for* in situ* analysis; (ii) enhancement of the performances by using nanotechnology-based modifications.

Among future trends of IVDDs, the employment of point-of-care (POC) devices will receive growing interest due to their characteristics of high accuracy and noninvasive devices also suitable for continuous monitoring. In particular, the POC tests will become an excellent choice for the diagnosis of infectious diseases in developing countries, mostly where those diseases are endemic, thanks to the possibility of being easily transported, installed, and also used by nonspecifically trained personnel. Also, to overcome issues of cost and improving performances of simple devices, it can be expected that configurations based on cheap substrates and readily available reader devices such as tablets or cellular phones will grow dramatically in the near future.

In fact, instead of focusing on enhancing the performance of the transduction system, the actual trend is more devoted towards addressing either nonspecific response or limitations of recognition-based reaction affinity. In this respect, a significant trend is the employment of aptamers instead of antibodies for the specific binding of proteins. Even if this technology has scored only a small number of successes so far, it will gain more attention as it entails the use of synthetically generated reagents in place of highly complex biomolecules obtained by living organisms and therefore often variable.

In our opinion, the synergic effect in the field of optics, nanotechnologies, microfluidics, biotechnologies, and surface chemistry will result in the development of improved SPR affinity biodevices which will be able to be used as an alternative to the traditional methods commonly employed for the* in vitro* diagnostics.

## Figures and Tables

**Table 1:** General classification of IVDD.

Class	Individual risk	Public health risk	Examples
A	*∙*	*∙*	Selective/differential microbiological media, identification kits for cultured microorganisms
B	*∙∙*	*∙*	Hormones, vitamins, enzymes, metabolic markers
C	*∙∙∙*	*∙∙*	PSA or CEA screening
D	*∙∙∙*	*∙∙∙*	HIV, HCV, HBV, HTLV

(*∙*) Low, (*∙∙*) moderate, and (*∙∙∙*) high.

**Table 2:** Main advantages and drawbacks about bulk SPR, localized SPR, and SPR imaging transduction in ABBs.

	Bulk SPR	Localized SPR	SPR imaging
*Advantages*			
Label-free	*∙∙*	*∙∙∙*	*∙∙∙*
Real-time monitoring	*∙∙∙*	*∙∙∙*	*∙∙∙*
Sensitivity	*∙*	*∙∙*	*∙∙∙*
Multidetection array	—	*∙∙*	*∙∙*
Reusability	*∙∙*	*∙∙*	*∙∙*
Being easy to perform	*∙∙*	*∙∙*	*∙∙*
Low sample/ligand volume required	*∙∙*	*∙∙∙*	*∙∙∙*

*Drawbacks*			
Steric hindrance	*∙∙*	*∙*	*∙*
Nonspecific adsorption of proteins	*∙∙*	*∙*	*∙*
Low molecular weight detection	*∙∙*	*∙*	*∙*

(*∙*) Low, (*∙∙*) moderate, and (*∙∙∙*) high.

**Table 3:** Main analytical performances of the reviewed biosensors based on bulk SPR.

Analytes	Nanoparticles	Biotransducer	Detection limit (LOD)	IVDD class	References
PSA	AuNPs	Antibody	0.1 ng mL^−1^	C	Jung et al., 2009 [32]
Ischemia modified albumin (IMA)	AuNPs	Antibody	10 ng mL^−1^	C	Li et al., 2013 [33]
Progesterone	AuNPs	Antibody	4.9 ng mL^−1^	B	Yuan et al., 2007 [34]
Insulin	AuNPs	Antibody	0.5 pM	B	Frasconi et al., 2010 [36]
Dopamine	AuNPs	Antibody	2 nM	B	Matsui et al., 2005 [37]
PSA	AuNPs	Antibody	0.3 ng mL^−1^	C	Uludag and Tothill, 2012 [38]
PSA	AuNPs	Antibody	300 fM	C	Choi et al., 2008 [39]
Human cardiac myoglobin (cMb)	AuNPs	Antibody	10 pM	B	Gnedenko et al., 2013 [40]
CEA	Bio-AuNPs	Antibody	0.1 ng mL^−1^	C	Špringer and Homola, 2012 [41]
CEA	AuNPs	Antibody	—	C	Špringer et al., 2014 [35]
Necrosis factor alpha (TNF-*α*)	Gold nanorods (GNRs)	Antibody	0.5 ng mL^−1^	C	Law et al., 2011 [42]
Thrombin (Thr)	AuNPs	Aptamer	0.1 nM	B	Bai et al., 2013 [43]
Brain natriuretic peptide (BNP)	Gold nanocubes (AuNCs)	Antibody/aptamer	—	C	Jang et al., 2014 [44]
Thrombin (Thr)	Nanorods (NRs) and quasi-spherical nanoparticles (qsNPs)	Aptamer	0.1 aM	B	Baek et al., 2014 [45]
Thrombin (Thr)	AuNPs	Aptamer	0.8 aM	B	He et al., 2014 [46]
Thrombin (Thr)	Fe_3_O_4_ MNPs	Aptamer	0.02 nM	B	Wang et al., 2011 [47]
*α*-fetoprotein (*α*-FET)	Core-shell Fe_3_O_4_-Au magnetic nanoparticles (MNPs)	Antibody	0.65 ng mL^−1^	C	Liang et al., 2012 [48]
Staphylococcal Enterotoxin B (SEB)	Immunomagnetic beads (IMBs)	Antibody	0.1 ng mL^−1^	C	Soelberg et al., 2009 [49]

**Table 4:** Main analytical performances of the reviewed biosensors based on localized SPR.

Analytes	Nanoparticles	Biotransducer	Detection limit (LOD)	IVDD class	References
Amyloid-derived diffusible ligands (ADDL)	AuNPs	Antibody	—	C	Haes et al., 2005 [50]
Avian influenza (A)	AuNPs	Antibody	1 pg mL^−1^	C	Park et al., 2012 [51]
HIV-1	AuNPs	Antibody	0.2 pg mL^−1^	D	Lee et al., 2013 [52]
Extracellular adherence protein (EAP)/PSA	AuNPs	Antibody	8 nM/1 pM	C	Chen et al., 2009 [53]
Squamous cell carcinoma antigen (SCCa)	AuNPs	Antibody	0.125 pM	C	Zhao et al., 2014 [54]
*α*-fetoprotein (AFP)/PSA	AuNPs	Antibody	41 zM	C	Aćimović et al., 2014 [55]
Human epididymis secretory protein 4 (HE4)	AgNPs	Antibody	4 pM	C	Yuan et al., 2012 [56]
Microalbuminuria	AuNPs	Antibody	1000 pg mL^−1^	C	Lai et al., 2010 [57]
p53 mutation	AgNPs	Antibody	10 nM	C	Duan et al., 2012 [58]
NADH	AuNPs	Antibody	—	B	Gu et al., 2015 [59]

**Table 5:** Main analytical performances of the reviewed biosensors based on SPR imaging.

Analytes	Nanoparticles	Biotransducer	Detection limit (LOD)	IVDD class	References
Nucleic Acid	AuNPs	Aptamer	1 fM	C	Sendroiu et al., 2011 [60]
miRNA	SiNPs	Aptamer	100 fM	C	Zhou et al., 2011 [61]
miRNA	AuNPs	Aptamer	5 aM	C	Fang et al., 2006 [62]
DNA	AuNPs	Aptamer	10 aM	C	Gifford et al., 2010 [63]
Single nucleotide polymorphisms (SNPs)	AuNPs	Aptamer	1 pM	C	Li et al., 2006 [14, 15]
Single nucleotide polymorphisms (SNPs)	AuNPs	Aptamer	32 nM	C	Sato et al., 2006 [64]
DNA	AuNPs	Aptamer	41 zM	C	D'Agata et al., 2010 [65]
DNA	AuNPs	Aptamer	0.02 aM	C	Yao et al., 2006 [66]
miRNA	AuNPs	Aptamer	—	C	Wood et al., 2013 [67]
IgG	AgNPs	Antibody	1 nM	B	Paul et al., 2011 [68]
